# Urine Drug Testing Among Long-term Opioid Prescribed Patients: Disparities and Opportunities

**DOI:** 10.1007/s40615-025-02474-x

**Published:** 2025-05-23

**Authors:** Brian K. Yorkgitis, Ira Harmon, Azad Khan, Fern Webb, Gabriel Brat

**Affiliations:** 1 Department of Surgery, Indiana University School of Medicine, 540 Barnhill Drive, Indianapolis, IN 46202 USA; 2 Department of Surgery, University of Florida College of Medicine-Jacksonville, 653 W. 8th Street, Jacksonville, FL 32209 USA; 3 Center for Data Solutions, University of Florida College of Medicine-Jacksonville, 653 W. 8th Street, Jacksonville, FL 32209 USA; 4 Department of Biomedical Informatics, Harvard Medical School, 10 Shattuck Street, Boston, MA 02115 USA

**Keywords:** Urine drug test, Opioid, Disparities, Pain, Long-term opioid use

## Abstract

**Background:**

There is wide variation in opioid prescribing practices, including opioid quantity and risk mitigation strategies (RMS). Urine drug tests (UDT) are often used as a RMS for patients prescribed opioids. There is a lack of standardized recommendations for these tests.

**Objective:**

We aim to evaluate differences in prescribing practices, including opioid prescriptions and UDT as an RMS, among patients with multiple opioid prescriptions.

**Design:**

A retrospective analysis of a national outpatient database of long-term adult opioid prescriptions (≥ 3 prescriptions over a period of at least 120-days) in the United States.

**Measurements:**

Demographics, abuse history, morphine milligram equivalents (MME), UDT, and frequency were variables of interest.

**Results:**

96,994 met the inclusion criteria. Hispanic patients were prescribed less MME/day than non-Hispanics. Examining patients prescribed ≥ 50 MME/day, the highest rates were in American Indian/Alaskan native (8.4%) and White patients (7.5%). At least one UDT was performed in 18,203 (18.8%) patients. When categorized by race, UDTs showed that 25.8% of American Indian/Alaska native, 22.7% of Black patients, 19.2% of multiple races, 18.0% of White patients, 13.5% of Hawaiian/Pacific Islanders, and 12.7% of Asian patients underwent UDTs (*p* < 0.001). Among the category of ≥ 7 UDTs, Black patients (1.3%) received the most. Upon regression modeling, females (OR 0.94) and uninsured patients (OR 0.66) were less likely to undergo UDT. Among MME categories, patients prescribed 75–99 MME/day had the highest likelihood of UDT (OR 2.4). Those with opioid use disorder (OR 2.64) and tobacco use (OR 1.083) were tested more frequently. When examining race, American Indian/Alaskan natives (OR 1.36), Black patients (OR 1.36), and multiple races (OR 1.245) were more likely to undergo UDT than White patients (reference).

**Conclusions:**

There is variation in opioid prescribing practices, including opioid quantity and UDT. White patients receive more opioids but less UDT than other populations. Improvements are needed to ensure universal opioid prescribing practices.

## Introduction

More than 131 million opioid prescriptions are dispensed each year in the United States [1]. To assist with the country’s opioid epidemic, mitigation strategies for opioid-associated risks call for “universal precautions” when managing long-term pain. These include making the correct pain diagnosis, performing a psychological risk assessment, obtaining informed consent and a treatment agreement, assessing and reassessing pain and function levels before and after interventions, beginning a trial of opioids with or without adjunctive therapies and medications, regularly assessing the “4 As” (analgesia, activity, adverse effects and aberrant behavior), periodically reviewing the pain diagnosis and co-occurring conditions, and documenting initial and follow-up visits [2].

While universal precautions aim to standardize care across all patients, disparities in opioid prescribing have been reported. Research shows significant differences in opioid prescription initiation and continuation across racial and ethnic groups [3]. For example, Black patients often receive fewer opioid prescriptions compared to White patients. One study found that Black patients discharged from inpatient services were less likely to receive opioid prescriptions and, when they did, were prescribed shorter courses than White patients [4]. Additionally, older Black patients reporting new lower back pain were prescribed fewer opioids than their White counterparts, with White patients receiving more opioid prescriptions than any other racial or ethnic group [5]. Even in the context of poor-prognosis cancer patients nearing the end of life, Black and Hispanic patients were prescribed lower doses of opioids compared to White patients [6].

Disparities extend beyond opioid prescriptions to risk mitigation strategies, such as urine drug testing (UDT), which is often used to assess compliance or aberrant behavior. In a 1-year retrospective study of patients on long-term opioid therapy (LTOT), only 39.6% of patients underwent UDT with significant variation. Black patients underwent UDT more frequently than other populations [7]. Among older patients with poor-prognosis cancer, Black patients underwent more UDT than other racial and ethnic groups [6]. Although local guidelines and regulations may endorse UDT, there is no national consensus on testing frequency, leaving significant variability at the discretion of individual prescribers; a practice shown to vary by race [8, 9].

Given the disparities, concerns that “universal precautions” are not universal, further investigation into opioid prescribing practices is warranted. Using a large, national retrospective outpatient population dataset, this study aims to examine urine drug testing and opioid prescribing patterns among patients who receive three or more opioid prescriptions for pain-provoking conditions. Building on previous findings of disparities in opioid prescribing and risk mitigation through UDT, we hypothesize that racial and ethnic minorities will receive fewer opioids but undergo more UDTs.

## Methods

We conducted a retrospective cohort study of patients with pain-provoking conditions that were prescribed at least three opioid prescriptions during a 10-year period using the OCHIN Accelerating Data Value Across a National Community Health Center Network (ADVANCE) data warehouse. To obtain data access, a data use agreement (DUA) was executed between all parties. The study was approved by the University of Florida and Harvard University Institutional Review Board as exempt.

### Study Sample

Patients included in the study were at least 18 years old, had a diagnosis of at least one pain-provoking condition and considered long-term opioid users, receiving three or more opioid prescriptions during a minimum of a 120-day period between January 1, 2012, and December 31, 2021. Given the data set time span, if patients had multiple minimum 120-day episodes, only the first episode was used for analysis. Patients were grouped into seven categories of pain-provoking conditions using International Classification of Diseases (ICD) 9 and 10 with the assistance of previous pain condition work done using OCHIN data: limb/extremity/joint arthritic disorders, back, urogenital/pelvic/menstrual, musculoskeletal chest, headache, and neck [10]. Patients with more than one condition within the opioid episode were placed in the “multiple” category. ICD-9 and 10 codes were used to determine inclusion in each category (Appendix 1). Institutionalized individuals, persons residing outside the United States and age < 18 years at the time of their first pain-provoking condition visit, were excluded in this analysis.

### Data Sources

The dataset was created by sampling retrospective de-identified data from the OCHIN ADVANCE data warehouse, which uses the Epic (Epic Systems Corporation, Madison, WI, USA) electronic health record (EHR) system. OCHIN is a national, nonprofit health IT consultancy that conducts healthcare equity research. The data warehouse aggregates electronic health records from over 6.3 million patients across more than 1600 clinics in over 30 states [11].

The use of OCHIN data requires adherence to their small cell size suppression policy (Policy 124_OCHIN_Research). This policy applies to research data shared with external partners and entities for the purpose of protecting patient confidentiality to minimize the likelihood of the identification of individual patients. Variables with patient counts containing a value of 1–10 cannot be reported or displayed. For any data point that is < 10, this is notated as “ < 10” and any associated values are reported as “ > or < X” to adhere to this patient privacy policy.

### Independent Variables

We assessed the following characteristics based on OCHIN record data: sociodemographic (as reported in the dataset): age (years), sex (female, male, other/unknown), race (White, American Indian/Alaskan native, Asian, Black/African American, Hawaiian/Pacific Islander, multiple, unknown), ethnicity (Hispanic, non-Hispanic), preferred language (English, non-English), and payor (private, Medicaid, Medicare, uninsured/other/unknown). For ethnicity, race, sex, and payor, similar categories were combined. For sex, the category “no information” was combined with “unknown”. For race and ethnicity, the categories “no information” and “X” were combined into “unknown.” For the insurer, “public” and “other/public” were combined with “Medicaid.” In the insurer category, “NA” was combined with “other/unknown.”

The dataset’s prescribed medication field was used to determine if patients were prescribed an opioid. Prescriptions are listed by name and identified by their RxNorm concept unique identifiers (RXCUI) and national drug code (NDC). To determine if a prescribed medication was an opioid, we first performed an inner join on the prescribing table with a second table that contained NDCs and RXCUIs, joining the variables. This joining was done because RXCUIs can represent multiple NDCs to assure accuracy when calculating the morphine milligram equivalent (MME). The patients’ prescribed medications were filtered for opioids identified by using DailyMed and RxNorm search for opioid medications [12]. Excluded drugs included diphenoxylate as it is commonly used for diarrhea, buprenorphine, and methadone, having both indications for medication-assisted treatment and pain management; reliable discernment of indication could not be achieved.

We then applied the NDCs’ of opioids identified in the prescribed medication list to those contained in the New York state MME conversion table [13]. This left only prescriptions for opioids and their MME which the prescribing information was used to calculate average daily MME. The names of medications on the resultant list were manually reviewed by a clinician for validation of included opioid medications.

To identify UDTs contained in the dataset, patient lab tests were queried for their logical observation identifiers, names, and codes (LOINC). The patient LOINC codes were cross-referenced with identified LOINC for UDTs of interest obtained through a search of the LOINC website LOINC v2.76 (LOINC.org Regenstrief Institute, Inc. Indianapolis, IN, USA). We excluded non-urine tests, barbiturates, nicotine, and alcohol. We augmented the original table with descriptions of tests that include the names of the drugs tested for.

The dataset was queried for diagnosis codes to determine if the patients had a history of alcohol (AUD) or opioid use disorder (OUD) or were currently using tobacco products (Appendix 1). Patients diagnosed with OUD or alcohol use disorder at any time prior to the start of their defined episode were considered positive for the corresponding diagnosis.

### Statistical Analysis

The dependent variable was urine drug testing, with several variables included in the analysis to identify differences based on patients’ health and sociodemographic characteristics. The primary outcome variable was the performance of UDT. The variable was defined as “Yes, had a UDT performed” during their defined opioid episode, or “No, never had a UDT performed” contained in their EHR information. If a UDT was performed, the number of UDTs was categorized into quantity clusters based on threes as a recommendation for high-risk patients to undergo three or more UDTs per year [14]. This variable was stratified by race. Covariates considered included age, sex, race, ethnicity, payor, daily MME, pain category, preferred language, history of AUD, history of OUD, and tobacco use.

Data were analyzed using R version 4.3.2 (R Projects, Vienna, Austria). We compared variables across groups using proportions and means. Multiple logistic regression was performed to develop odds ratios (OR). Effect sizes and 95% confidence intervals (CIs) are reported along with *p*-values. The level of significance was set at < 0.05.

## Results

This population data source included 96,994 patients who met the inclusion criteria. The cohort had 58,246 (60.0%) females and 38,725 (40.0%) males. The mean age was 53.1 ± 14.1 years. The majority preferred the English language for healthcare delivery (90.4%). Pain conditions, in order of frequency, were multiple, limb/extremity, back, neck, urogenital/pelvic, musculoskeletal chest, and headache. Medicaid (39.4%) followed by Medicare (33.4%) were the most common insurance types. Private insurance accounted for 11.7% (Table 1).

**Table 1:** Patient characteristics and urine drug testing (UDT)

	Overall	No UDT	UDT	*p*-value
	*N* = 96,994	*N* = 78,791	*N* = 18,203	
Age (years)				< 0.001
18–20	352	325 (92%)	27 (7.7%)	
21–30	5879	4892 (83%)	987 (17%)	
31–40	12,812	10,322 (81%)	2490 (19%)	
41–50	19,679	15,671 (80%)	4008 (20%)	
51–60	29,999	23,867 (80%)	6132 (20%)	
61–70	18,516	15,030 (81%)	3486 (19%)	
71–80	6745	5883 (87%)	862 (13%)	
81–90	2544	2345 (92%)	199 (7.8%)	
91 +	468	456 (97%)	12 (2.6%)	
Sex				< 0.001
Female	58,246	> 47,000 (> 80%)*	> 10,000 (> 15%)*	
Male	38,725	> 30,000 (> 75%)*	> 7000 (> 15%)*	
Other/unknown	< 30*	18 (> 70%)*	< 10*	
Race				< 0.001
American Indian\Alaska native	1249	927 (74%)	322 (26%)	
Asian	1407	1229 (87%)	178 (13%)	
Black/African American	15,097	11,677 (77%)	3420 (23%)	
Hawaiian/Pacific Islander	520	450 (87%)	70 (13%)	
Multiple	1253	1012 (81%)	241 (19%)	
Unknown	4175	3412 (82%)	763 (18%)	
White	73,293	60,084 (82%)	13,209 (18%)	
Hispanic				< 0.001
No	82,446	66,234 (80%)	16,212 (20%)	
Yes	14,548	12,557 (86%)	1991 (14%)	
English preferred				< 0.001
No	9286	8402 (90%)	884 (9.5%)	
Yes	87,708	70,389 (80%)	17,319 (20%)	
Payor				< 0.001
Medicaid	44,329	34,761 (78%)	9568 (22%)	
Medicare	26,974	21,552 (80%)	5422 (20%)	
Other/unknown	66	66 (100%)	0 (0%)	
Private	11,724	9959 (85%)	1765 (15%)	
Uninsured	13,901	12,453 (90%)	1448 (10%)	
Tobacco use				< 0.001
No	56,288	46,205 (82%)	10,083 (18%)	
Yes	40,706	32,586 (80%)	8120 (20%)	
History of AUD				0.009
No	93,586	76,082 (81%)	17,504 (19%)	
Yes	3408	2709 (79%)	699 (21%)	
History of OUD				< 0.001
No	94,787	77,568 (82%)	17,219 (18%)	
Yes	2207	1223 (55%)	984 (45%)	
Pain cluster				< 0.001
Back	15,004	12,089 (81%)	2915 (19%)	
Headache	773	670 (87%)	103 (13%)	
Limb/extremity/joint/arthritic	21,338	17,642 (83%)	3696 (17%)	
Multiple	56,329	45,424 (81%)	10,905 (19%)	
Musculoskeletal chest	828	724 (87%)	104 (13%)	
Neck	1829	1438 (79%)	391 (21%)	
Urogenital/pelvic/menstrual	893	804 (90%)	89 (10.0%)	
Average daily MME				< 0.001
≤ 24	73,697	62,105 (84%)	11,592 (16%)	
25–49	16,480	11,874 (72%)	4606 (28%)	
50–74	3660	2,629 (72%)	1031 (28%)	
75–99	1371	914 (67%)	457 (33%)	
99 +	1786	1269 (71%)	517 (29%)	

*OUD* opioid use disorder, *AUD* alcohol use disorder, *MME* morphine milligram equivalent

Significance set at *p* < 0.05

*Value cannot be disclosed due to cell size suppression policy

Documented OUD was present in 2.3% of the population, highest in the American Indian/Alaskan native populations (3.3%). Tobacco use was present in 42% of the cohort, with the highest prevalence among those of multiple races (47%). Alcohol use disorder was documented in 3.5% of patients, with American Indian/Alaskan native populations being the highest at 45% (Table 1).

When examining opioid prescriptions daily MME, there were differences among racial and ethnic groups (*p* < 0.001 for both race and ethnicity). Hispanic patients were prescribed less MME/day than non-Hispanics. When examining racial categories receiving ≥ 50 MME/day, American Indian/Alaskan natives (8.4%) and White patients (7.5%) had the highest rates. Asian patients (2.4%) had the lowest, followed by Hawaiian/Pacific Islander (4.9%), Black/African American (5.1%), and multiple races (5.4%) for receipt of a prescription ≥ 50 MME/day (Table 2).

**Table 2:** Average daily MME by race and ethnicity

	Average daily MME							
	Overall *N* = 96,994	< 24 MME	25–49 MME	50–7 4MME	75–99 MME	99 + MME	Total > 50 MME	*p*-value
		*N* = 73,697	*N* = 16,480	*N* = 3660	*N* = 1371	*N* = 1786	*N* = 6817	
Hispanic ethnicity								< 0.001
No	82,446 (100%)	61,294 (74%)	14,839 (18%)	3382 (4.1%)	1274 (1.5%)	1657 (2.0%)	6313 (7.7%)	
Yes	14,548 (100%)	12,403 (85%)	1,641 (11%)	278 (1.9%)	97 (0.7%)	129 (0.9%)	504 (3.5%)	
Race								< 0.001
American Indian/Alaska native	1249 (100%)	916 (73%)	228 (18%)	54 (4.3%)	26 (2.1%)	25 (2.0%)	105 (8.4%)	
Asian	1407 (100%)	1229 (87%)	144 (10%)	21 (1.5%)	<10(<1.0%)*	<10 (<1.0%)*	34 (2.4%)	
Black/African American	15,097 (100%)	11,898 (79%)	2374 (16%)	429 (2.8%)	180 (1.2%)	216 (1.4%)	825 (5.5%)	
Hawaiian/Pacific Islander	520 (100%)	421 (81%)	74 (14%)	17 (3.3%)	<10 (<1.0%)*	<10(<2.01%)*	25 (4.9%)	
Multiracial	1253 (100%)	964 (77%)	204 (16%)	42 (3.4%)	17 (1.4%)	26 (2.1%)	85 (6.8%)	
Unknown/other	4175 (100%)	3322 (80%)	626 (15%)	138 (3.3%)	43 (1.0%)	46 (1.1%)	227 (5.4%)	
White	73,293 (100%)	54,947 (75%)	12,830 (18%)	2959 (4.0%)	1096 (1.5%)	1461 (2.0%)	5516 (7.5%)	

*MME* morphine milligram equivalents

*Value cannot be disclosed due to cell size suppression policy

Significance set at *p* < 0.05

Regarding UDT as the outcome, 18,203 (18.8%) patients that were prescribed three or more opioid prescriptions had at least one test. Among races, 25.8% of American Indian/Alaska natives, 22.7% of Black patients, 19.2% of multiple races, 18.0% of White patients, 13.5% of Hawaiian/Pacific Islanders, and 12.7% of Asians were tested. Among MME clusters, those prescribed over 25 MME/day received UDT more than those below. The number of UDTs per patient showed 86.4% had 1–3 UDTs, 8.5% had 4–6 UDTs, and 5% had ≥ 7 UDTs. In each of these numbers of UDTs categories, Asian patients were tested the least. There were differences in each number of UDTs among different races (*P* < 0.001). Among the highest category of UDTs (≥ 7), Black patients (1.3%) and Hawaiian/Pacific Islander patients received the most, followed by American Indian/Alaska native patients (1.0%), White patients and unknown patients (both 0.9%), and lastly, Asian patients (Table 3).

**Table 3:** Urine drug test (UDT) by race and ethnicity

	Overall	No UDT	1–3 UDTs	4–6 UDTs	7 + UDTs	*p*-value
	*N* = 96,994	N = 78,791	*N* = 15,727	*N* = > 1500*	*N* = > 900*	
Hispanic						< 0.001
Yes	14,548	12,557	1602	213	176	
No	82,446	66,234	14,125	1353	734	
Race						< 0.001
American Indian/Alaska native	1249	927 (74%)	264 (21%)	43 (> 3%)*	15 (1.2%)	
Asian	1407	1229 (87%)	158 (11%)	11 (< 1%)*	< 10*	
Black/African American	15,097	11,677 (77%)	2903 (19%)	319 (> 2%)*	198 (1.3%)	
Hawaiian/Pacific Islander	520	450 (87%)	58 (11%)	< 10*	< 10*	
Multiracial	1253	1012 (81%)	196 (16%)	32 (> 2%)*	13 (1.0%)	
Unknown/other	4175	3412 (82%)	658 (16%)	67 (> 1%)*	38 (0.9%)	
White	73,293	60,084 (82%)	11,490 (16%)	1,089 (> 1%)*	630 (0.9%)	

*UDT* urine drug test

*Value cannot be disclosed due to cell size suppression policy, significance set at *p* < 0.05

Upon multivariate logistical regression, several factors were found to influence UDT. Female patients (OR 0.94) were less likely to undergo UDT. Ages 21 to 70 are more likely to undergo UDT than those patients < 21 years (reference group). Patients prescribed an average of 75–99 MME per day had the highest rate of a UDT (OR 2.4 CI) among daily MME clusters. Uninsured patients (OR 0.66) were less likely to receive a UDT. Patients with AUD were tested less (OR 0.86) and those with OUD (OR 2.64) and tobacco use (OR 1.08) were tested more. Regarding race, American Indian/Alaskan natives (OR 1.36), Black patients (OR 1.36), patients of multiple races (OR 1.25), and those of unknown race (OR 1.12) were more likely to undergo UDT compared to White patients (reference group). Hispanic patients’ UDT was not different from that of non-Hispanic patients. Patients who preferred to receive their healthcare in the English language (OR 1.75) had an increase in receipt of a UDT (Table 4).

**Table 4:** Regression modeling for urine drug testing

	Odds ratio	CI—95% (lower)	CI—95% (upper)	*P*-value
Age (years)				
18–20 (reference)	**1**	**–-**	**–-**	**–-**
21–30	2.321	1.552	3.473	< 0.001
31–40	2.699	1.81	4.023	< 0.001
41–50	2.806	1.884	4.18	< 0.001
51–60	2.688	1.805	4.002	< 0.001
61–70	2.32	1.556	3.458	< 0.001
71–80	1.472	0.982	2.207	0.062
81–90	0.894	0.585	1.367	0.606
91 +	0.284	0.141	0.573	< 0.001
Sex				
Male (reference)	**1**	**–-**	**–-**	**–-**
Female	0.922	0.891	0.954	< 0.001
Other/unknown	0.928	0.337	2.555	0.885
Hispanic				
No (reference)	**1**	**–-**	**–-**	**–-**
Yes	0.894	0.844	0.948	< 0.001
Race				
White (reference)	**1**	**–-**	**–-**	**–-**
American Indian/Alaska native	1.373	1.203	1.566	< 0.001
Asian	0.91	0.772	1.074	0.265
Black/African American	1.375	1.314	1.438	< 0.001
Hawaiian/Pacific Islander	0.658	0.508	0.851	0.001
Multiple	0.954	0.824	1.103	0.523
Unknown	1.166	1.072	1.269	< 0.001
Payor				
Private (reference)	**1**	**–-**	**–-**	**–-**
Medicaid	1.58	1.492	1.673	< 0.001
Medicare	1.566	1.471	1.668	< 0.001
Uninsured/other/unknown	0.699	0.648	0.754	< 0.001
English preferred				
No (reference)	**1**	**–-**	**–-**	**–-**
Yes	1.732	1.596	1.879	< 0.001
Tobacco use				
No (reference)	**1**	**–-**	**–-**	**–-**
Yes	0.994	0.96	1.03	0.747
History AUD				
No (reference)	**1**	**–-**	**–-**	**–-**
Yes	0.892	0.816	0.974	0.011
History of OUD				
No (reference)	**1**	**–-**	**–-**	**–-**
Yes	2.821	2.411	3.082	< 0.001
Pain cluster				
Back (reference)	**1**	**–-**	**–-**	**–-**
Headache	0.788	0.632	0.982	0.034
Limb/extremity/joint/arthritic	0.894	0.845	0.945	< 0.001
Multiple	0.97	0.925	1.017	0.209
Musculoskeletal chest	0.64	0.516	0.794	< 0.001
Neck	1.065	0.941	1.206	0.317
Urogenital/pelvic/menstrual	0.514	0.409	0.646	< 0.001
Average daily MME				
≤ 24 (reference)	**1**	**–-**	**–-**	**–-**
25–49	1.9	1.824	1.979	< 0.001
50–74	1.887	1.747	2.038	< 0.001
75–99	2.358	2.096	2.653	< 0.001
99 +	1.896	1.703	2.111	< 0.001

*OUD* opioid use disorder, *AUD* alcohol use disorder, *MME* morphine milligram equivalent

Significance set at *p* < 0.05

## Discussion

Rates of UDT in previous literature ranged from 2 to 50% among opioid-prescribed patients [15]. In this outpatient cohort of pain-provoking conditions who were prescribed at least three opioid prescriptions, 18.8% received at least one UDT. This study adds to the literature that disparities not only exist in opioid prescribing but also in the risk mitigation practice of UDT. While White patients received more MME on average per day, they were less likely to undergo UDT than non-white patients, with the exception of Asian and Hawaiian/Pacific Islander patients. Asian and Pacific Islander patients received fewer opioids than other racial groups, consistent with previous studies, which may account for their lower UDT rate [5]. When UDT was performed, White patients were tested less frequently than Black patients, raising concerns about potential bias in UDT practices. This highlights the need for evidenced-based UDT screening protocols that are universally applied.

UDT among long-term opioid patients has increased over time, doubling between the years 2012 and 2018. A large national commercial insurance database study found that patients in the South, as well as those with back pain or other chronic pain conditions, were more likely to be tested [15]. However, the study did not account for important variables such as race or ethnicity. Without clear guidance regarding the time intervals or number of UDTs to be performed, testing is often determined by local practice patterns and provider discretion [8, 9]. With this, discretion comes the risk of subjective practices influenced by patient demographics. Our study expands on the literature by examining differences in UDT practices across racial groups.

Another consideration in UDT is the cost burden, not only to the healthcare system but also to the patients [9]. It is estimated that UDT accounts for approximately $8.5 billion in costs per year [16]. As cost sharing increases for patients and Medicaid eligibility decreases, the financial burden of UDTs often falls on the patient. If patients are unable to afford testing, they may risk discontinuation of opioid therapy, which is often determined by the prescriber’s discretion or protocol. Data show that abrupt discontinuation of prescribed opioid therapy can lead to harm and illicit opioid use [17, 18]. Given the disparities found in UDT practices within this population, minority patients face burdens that extend beyond the test itself. Ensuring that the right patient receives the right test at the right time, based on evidence, is critical for healthcare stewardship and equity. In this study, uninsured patients were less likely to undergo UDT, which could be reflective of the cost sensitivity of these tests. However, it introduces additional concerns about the lack of universal precautions needed for risk mitigation. Local regulations have been enacted over recent years that have varying requirements for UDT, which may create more uniformity in that location. However, there is substantial variation and interpretation of these regulations for each locality [19].

When examining healthcare databases, several limitations should be acknowledged. Granular encounter data, ensuring all diagnoses are captured, conditions, and prescribed medications and UDT that may occur outside the EHR could be a limitation. However, these limitations are likely minimized by the extensive data network captured by OCHIN. Previous work has shown the OCHIN data is more comprehensive than Medicaid claims data, especially among populations that are discontinuous insurance coverage [20, 21]. Each prescriber or practice may have its own guidelines for opioid risk mitigation. Understanding provider-level decisions is beyond the scope of this dataset. These guidelines or practice patterns could include the use of a validated opioid risk screening tool that considers factors such as family history of substance abuse, sexual abuse, psychological disease, and various self-reported questionnaires—information beyond the data examined [22]. Additionally, local regulations enacted during the study period may have influenced opioid prescribing patterns, including UDT practices; however, the wide variation in these regulations limits our ability to examine them thoroughly in this dataset [19]. However, given the large national population, this limitation is likely mitigated. Socioeconomic factors are an important variable in this population. While the dataset contains federal poverty level (FPL) information, the high missing variable rate cannot be meaningfully incorporated into the analysis. Insurance status was used as a surrogate.

## Conclusion

There remains significant variation in opioid prescribing practices among different populations. UDT is more prevalent among minority populations within this outpatient dataset in receipt of three or more opioid prescriptions. While the term “universal precautions” is promoted to guide opioid prescribing in pain management, ensuring true universality across all populations is necessary to prevent disparities in risk, not only prescribing but also risk mitigation practices. Further research is needed to develop evidence-based protocols for equitable opioid prescribing and utilization of UDTs to ensure these practices are genuinely universal.

## Data Availability

The ADVANCE Data Warehouse includes patient-level data generated from multiple agencies and institutions; restrictions apply to the availability and re-release of data under cross-institution agreements. In addition, all clinical data that was used in this study exist within patient charts and were not collected as part of this study. Therefore, to honor member/partner agreements and protect potentially identifiable patient information, patient-level datasets, Epic code, and variable names cannot be shared publicly.

## Appendix

Table 5.

**Table 5:** Condition definitions

	ICD-10-CM
Back pain	G54.1, G54.3, G54.4, G96.11, L40.53, M08.1, M25.78, M43.00, M43.04, M43.05, M43.06, M43.07, M43.08, M43.09, M43.10, M43.14, M43.15, M43.16, M43.17, M43.18, M43.19, M43.5X4, M43.5X5, M43.5X6, M43.5X7, M43.5X8, M43.5X9, M43.8X4, M43.8X5, M43.8X6, M43.8X7, M43.8X8, M43.8X9, M43.9, M45.0, M45.4, M45.5, M45.6, M45.7, M45.8, M45.9, M46.00, M46.04, M46.05, M46.06, M46.07, M46.08, M46.09, M46.1, M46.40, M46.44, M46.45, M46.46, M46.47, M46.48, M46.49, M46.80, M46.84, M46.85, M46.86, M46.87, M46.88, M46.89, M46.90, M46.94, M46.95, M46.96, M46.97, M46.98, M46.99, M47.10, M47.14, M47.15, M47.16, M47.20, M47.24, M47.25, M47.26, M47.27, M47.28, M47.814, M47.815, M47.816, M47.817, M47.818, M47.819, M47.894, M47.895, M47.896, M47.897, M47.898, M47.899, M47.9, M48.00, M48.04, M48.05, M48.06, M48.07, M48.08, M48.10, M48.14, M48.15, M48.16, M48.17, M48.18, M48.19, M48.20, M48.24, M48.25, M48.26, M48.27, M48.30, M48.34, M48.35, M48.36, M48.37, M48.38, M48.8X4, M48.8X5, M48.8X6, M48.8X7, M48.8X8, M48.8X9, M48.9, M49.80, M49.84, M49.85, M49.86, M49.87, M49.88, M49.89, M51.04, M51.05, M51.06, M51.14, M51.15, M51.16, M51.17, M51.24, M51.25, M51.26, M51.27, M51.34, M51.35, M51.36, M51.37, M51.44, M51.45, M51.46, M51.47, M51.84, M51.85, M51.86, M51.87, M51.9, M53.2X4, M53.2X5, M53.2X6, M53.2X7, M53.2X8, M53.2X9, M53.3, M53.80, M53.84, M53.85, M53.86, M53.87, M53.88, M53.9, M54.00, M54.04, M54.05, M54.06, M54.07, M54.08, M54.09, M54.10, M54.14, M54.15, M54.16, M54.17, M54.18, M54.30, M54.31, M54.32, M54.40, M54.41, M54.42, M54.5, M54.6, M54.89, M54.9, M62.830, M95.4, M95.5, M96.1, M99.02, M99.03, M99.04, M99.05, M99.12, M99.14, M99.18, M99.53, M99.73, M99.79, M99.82, M99.83, M99.84, M99.85, M99.88, Q67.5, Q76.0, Q76.1, Q76.2, Q76.3, Q76.414, Q76.415, Q76.419, Q76.49, S23.100 A, S23.101 A, S23.101D, S23.101S, S23.111 A, S23.111D, S23.111S, S23.121 A, S23.121D, S23.121S, S23.123 A, S23.123D, S23.123S, S23.131 A, S23.131D, S23.131S, S23.133 A, S23.133D, S23.133S, S23.141 A, S23.141D, S23.141S, S23.143 A, S23.143D, S23.143S, S23.151 A, S23.151D, S23.151S, S23.153 A, S23.153D, S23.153S, S23.161 A, S23.161D, S23.161S, S23.163 A, S23.163D, S23.163S, S23.171 A, S23.171D, S23.171S, S23.20XA, S23.20XD, S23.20XS, S23.29XA, S23.29XD, S23.29XS, S23.3XXA, S23.3XXD, S23.3XXS, S23.8XXA, S23.8XXD, S23.8XXS, S23.9XXA, S23.9XXD, S23.9XXS, S29.019 A, S29.019D, S29.019S, S33.0XXA, S33.100 A, S33.101 A, S33.101D, S33.101S, S33.111 A, S33.111D, S33.111S, S33.121 A, S33.121D, S33.121S, S33.131 A, S33.131D, S33.131S, S33.140 A, S33.140D, S33.141 A, S33.141D, S33.141S, S33.2XXA, S33.2XXD, S33.2XXS, S33.30XA, S33.30XD, S33.30XS, S33.39XA, S33.39XD, S33.39XS, S33.5XXA, S33.5XXD, S33.5XXS, S33.6XXA, S33.6XXD, S33.6XXS, S33.8XXA, S33.8XXD, S33.8XXS, S33.9XXA, S33.9XXD, S33.9XXS, S39.012 A, S39.012D, S39.012S, S39.92XA
Neck pain	G54.2, M43.01, M43.02, M43.03, M43.11, M43.12, M43.13, M43.3, M43.4, M43.5X2, M43.5X3, M43.6, M43.8X1, M43.8X2, M43.8X3, M45.1, M45.2, M45.3, M46.01, M46.02, M46.03, M46.41, M46.42, M46.43, M46.81, M46.82, M46.83, M46.91, M46.92, M46.93, M47.11, M47.12, M47.13, M47.22, M47.811, M47.812, M47.813, M47.891, M47.892, M47.893, M48.01, M48.02, M48.03, M48.11, M48.12, M48.13, M48.21, M48.22, M48.23, M48.31, M48.32, M48.33, M48.8X1, M48.8X2, M48.8X3, M49.81, M49.82, M49.83, M50.00, M50.01, M50.020, M50.021, M50.022, M50.023, M50.03, M50.10, M50.11, M50.120, M50.121, M50.122, M50.123, M50.13, M50.20, M50.21, M50.220, M50.221, M50.222, M50.223, M50.23, M50.30, M50.31, M50.320, M50.321, M50.322, M50.323, M50.33, M50.80, M50.81, M50.820, M50.821, M50.822, M50.823, M50.83, M50.90, M50.91, M50.920, M50.921, M50.922, M50.923, M50.93, M53.0, M53.1, M53.2X1, M53.2X2, M53.2X3, M53.81, M53.82, M53.83, M54.01, M54.02, M54.03, M54.11, M54.12, M54.13, M54.2, M95.3, M99.01, M99.11, M99.31, M99.51, M99.61, M99.71, M99.81, Q76.411, Q76.412, Q76.413, S13.0XXA, S13.100 A, S13.100D, S13.101 A, S13.101D, S13.101S, S13.111 A, S13.111D, S13.111S, S13.120 A, S13.121 A, S13.121D, S13.121S, S13.130 A, S13.131 A, S13.131D, S13.131S, S13.140 A, S13.141 A, S13.141D, S13.141S, S13.150 A, S13.151 A, S13.151D, S13.151S, S13.160 A, S13.160D, S13.161 A, S13.161D, S13.161S, S13.170D, S13.171 A, S13.171D, S13.171S, S13.180 A, S13.181 A, S13.181D, S13.181S, S13.20XA, S13.20XD, S13.20XS, S13.29XA, S13.29XD, S13.29XS, S13.4XXA, S13.4XXD, S13.4XXS, S13.5XXA, S13.8XXA, S13.8XXD, S13.8XXS, S13.9XXD, S13.9XXS, S16.1XXA, S16.1XXD, S16.1XXS
Limb/extremity pain, joint pain, and arthritic disorders	A52.16, G54.0, G54.5, G54.6, G56.00, G56.01, G56.02, G56.03, G56.40, G56.41, G56.42, G56.43, G56.2, G57.00, G57.01, G57.02, G57.03, G57.10, G57.11, G57.12, G57.13, G57.20, G57.21, G57.22, G57.23, G57.30, G57.31, G57.32, G57.33, G57.40, G57.41, G57.42, G57.43, G57.50, G57.51, G57.52, G57.53, G57.60, G57.61, G57.62, G57.63, G57.70, G57.71, G57.72, G57.73, G72.9, I77.6, M02.00, M02.011, M02.012, M02.019, M02.021, M02.022, M02.029, M02.031, M02.032, M02.039, M02.041, M02.042, M02.049, M02.051, M02.052, M02.059, M02.061, M02.062, M02.069, M02.071, M02.072, M02.079, M02.08, M02.09, M06.219, M07.60, M07.611, M07.612, M07.619, M07.621, M07.622, M07.629, M07.631, M07.632, M07.639, M07.641, M07.642, M07.649, M07.651, M07.652, M07.659, M07.661, M07.662, M07.669, M07.671, M07.672, M07.679, M07.68, M07.69, M10.00, M10.011, M10.012, M10.019, M10.021, M10.022, M10.029, M10.031, M10.032, M10.039, M10.041, M10.042, M10.049, M10.051, M10.052, M10.059, M10.061, M10.062, M10.069, M10.071, M10.072, M10.079, M10.08, M10.09, M10.10, M10.172, M10.179, M10.20, M10.221, M10.241, M10.242, M10.261, M10.262, M10.269, M10.271, M10.272, M10.279, M10.30, M10.311, M10.312, M10.319, M10.321, M10.322, M10.329, M10.331, M10.332, M10.339, M10.341, M10.342, M10.349, M10.351, M10.352, M10.359, M10.361, M10.362, M10.369, M10.371, M10.372, M10.379, M10.38, M10.39, M10.40, M10.411, M10.412, M10.419, M10.421, M10.422, M10.429, M10.431, M10.432, M10.439, M10.441, M10.442, M10.449, M10.451, M10.452, M10.459, M10.461, M10.462, M10.469, M10.471, M10.472, M10.479, M10.48, M10.49, M10.9, M11.162, M11.20, M11.211, M11.212, M11.219, M11.221, M11.222, M11.229, M11.231, M11.232, M11.239, M11.241, M11.242, M11.249, M11.251, M11.252, M11.259, M11.261, M11.262, M11.269, M11.271, M11.272, M11.279, M11.28, M11.29, M11.80, M11.811, M11.812, M11.819, M11.821, M11.822, M11.829, M11.831, M11.832, M11.839, M11.841, M11.842, M11.849, M11.851, M11.852, M11.859, M11.861, M11.862, M11.869, M11.871, M11.872, M11.879, M11.88, M11.89, M11.9, M12.00, M12.011, M12.012, M12.019, M12.021, M12.022, M12.029, M12.031, M12.032, M12.039, M12.041, M12.042, M12.049, M12.051, M12.052, M12.059, M12.061, M12.062, M12.069, M12.071, M12.072, M12.079, M12.08, M12.09, M12.50, M12.511, M12.512, M12.519, M12.521, M12.522, M12.529, M12.531, M12.532, M12.539, M12.541, M12.542, M12.549, M12.551, M12.552, M12.559, M12.561, M12.562, M12.569, M12.571, M12.572, M12.579, M12.58, M12.59, M12.80, M12.811, M12.812, M12.819, M12.821, M12.822, M12.829, M12.831, M12.832, M12.839, M12.841, M12.842, M12.849, M12.851, M12.852, M12.859, M12.861, M12.862, M12.869, M12.871, M12.872, M12.879, M12.88, M12.89, M12.9, M13.0, M13.10, M13.111, M13.112, M13.119, M13.121, M13.122, M13.129, M13.131, M13.132, M13.139, M13.141, M13.142, M13.149, M13.151, M13.152, M13.159, M13.161, M13.162, M13.169, M13.171, M13.172, M13.179, M13.80, M13.811, M13.812, M13.819, M13.821, M13.822, M13.829, M13.831, M13.832, M13.839, M13.841, M13.842, M13.849, M13.851, M13.852, M13.859, M13.861, M13.862, M13.869, M13.871, M13.872, M13.879, M13.88, M13.89, M14.60, M14.611, M14.612, M14.619, M14.621, M14.622, M14.629, M14.631, M14.632, M14.639, M14.641, M14.642, M14.649, M14.651, M14.652, M14.659, M14.661, M14.662, M14.669, M14.671, M14.672, M14.679, M14.68, M14.69, M15.0, M15.1, M15.2, M15.3, M15.4, M15.8, M15.9, M16.0, M16.10, M16.11, M16.12, M16.2, M16.30, M16.31, M16.32, M16.4, M16.50, M16.51, M16.52, M16.6, M16.7, M16.9, M17.0, M17.10, M17.11, M17.12, M17.2, M17.30, M17.31, M17.32, M17.4, M17.5, M17.9, M18.0, M18.10, M18.11, M18.12, M18.2, M18.30, M18.31, M18.32, M18.4, M18.50, M18.51, M18.52, M18.9, M19.011, M19.012, M19.019, M19.021, M19.022, M19.029, M19.031, M19.032, M19.039, M19.041, M19.042, M19.049, M19.071, M19.072, M19.079, M19.111, M19.112, M19.119, M19.121, M19.122, M19.129, M19.131, M19.132, M19.139, M19.141, M19.142, M19.149, M19.171, M19.172, M19.179, M19.211, M19.212, M19.219, M19.221, M19.222, M19.229, M19.231, M19.232, M19.239, M19.241, M19.242, M19.249, M19.271, M19.272, M19.279, M19.90, M19.91, M19.92, M19.93, M1 A.00X0, M1 A.00X1, M1 A.0110, M1 A.0111, M1 A.0120, M1 A.0121, M1 A.0190, M1 A.0191, M1 A.0210, M1 A.0211, M1 A.0220, M1 A.0221, M1 A.0290, M1 A.0291, M1 A.0310, M1 A.0311, M1 A.0320, M1 A.0321, M1 A.0390, M1 A.0391, M1 A.0410, M1 A.0411, M1 A.0420, M1 A.0421, M1 A.0490, M1 A.0491, M1 A.0510, M1 A.0511, M1 A.0520, M1 A.0521, M1 A.0590, M1 A.0591, M1 A.0610, M1 A.0611, M1 A.0620, M1 A.0621, M1 A.0690, M1 A.0691, M1 A.0710, M1 A.0711, M1 A.0720, M1 A.0721, M1 A.0790, M1 A.0791, M1 A.08X0, M1 A.08X1, M1 A.09X0, M1 A.09X1, M1 A.10X0, M1 A.1421, M1 A.1690, M1 A.1720, M1 A.1790, M1 A.20X0, M1 A.2211, M1 A.2720, M1 A.29X0, M1 A.30X0, M1 A.30X1, M1 A.3320, M1 A.3421, M1 A.3490, M1 A.3491, M1 A.3710, M1 A.3711, M1 A.3720, M1 A.3721, M1 A.3790, M1 A.39X0, M1 A.39X1, M1 A.40X0, M1 A.40X1, M1 A.4490, M1 A.4710, M1 A.4720, M1 A.4791, M1 A.49X0, M1 A.49X1, M1 A.9XX0, M1 A.9XX1, M21.611, M21.612, M21.619, M22.01, M22.02, M22.10, M22.11, M22.12, M22.40, M22.41, M22.42, M23.50, M23.51, M23.52, M24.10, M24.111, M24.112, M24.119, M24.121, M24.122, M24.129, M24.131, M24.132, M24.139, M24.141, M24.142, M24.149, M24.151, M24.152, M24.159, M24.171, M24.172, M24.173, M24.174, M24.175, M24.176, M24.20, M24.211, M24.212, M24.219, M24.221, M24.222, M24.229, M24.231, M24.232, M24.239, M24.241, M24.242, M24.249, M24.251, M24.252, M24.259, M24.271, M24.272, M24.273, M24.274, M24.275, M24.276, M24.28, M24.40, M24.411, M24.412, M24.419, M24.421, M24.422, M24.429, M24.431, M24.432, M24.439, M24.441, M24.442, M24.443, M24.444, M24.445, M24.446, M24.451, M24.452, M24.459, M24.461, M24.462, M24.469, M24.471, M24.472, M24.473, M24.474, M24.475, M24.476, M24.477, M24.478, M24.479, M24.50, M24.511, M24.512, M24.519, M24.521, M24.522, M24.529, M24.531, M24.532, M24.539, M24.541, M24.542, M24.549, M24.551, M24.552, M24.559, M24.561, M24.562, M24.569, M24.571, M24.572, M24.573, M24.574, M24.575, M24.576, M24.60, M24.611, M24.612, M24.619, M24.621, M24.622, M24.629, M24.631, M24.632, M24.639, M24.641, M24.642, M24.649, M24.651, M24.652, M24.659, M24.661, M24.662, M24.669, M24.671, M24.672, M24.673, M24.674, M24.675, M24.676, M24.7, M25.50, M25.511, M25.512, M25.519, M25.521, M25.522, M25.529, M25.531, M25.532, M25.539, M25.541, M25.542, M25.549, M25.551, M25.552, M25.559, M25.561, M25.562, M25.569, M25.571, M25.572, M25.579, M25.70, M25.711, M25.712, M25.719, M25.721, M25.722, M25.729, M25.731, M25.732, M25.739, M25.741, M25.742, M25.749, M25.751, M25.752, M25.759, M25.761, M25.762, M25.769, M25.771, M25.772, M25.773, M25.774, M25.775, M25.776, M35.4, M62.40, M62.411, M62.412, M62.419, M62.421, M62.422, M62.429, M62.431, M62.432, M62.439, M62.441, M62.442, M62.449, M62.451, M62.452, M62.459, M62.461, M62.462, M62.469, M62.471, M62.472, M62.479, M62.48, M62.49, M62.831, M62.838, M62.89, M65.00, M65.011, M65.012, M65.019, M65.021, M65.022, M65.029, M65.031, M65.032, M65.039, M65.041, M65.042, M65.049, M65.051, M65.052, M65.059, M65.061, M65.062, M65.069, M65.071, M65.072, M65.079, M65.08, M65.10, M65.161, M65.20, M65.221, M65.222, M65.229, M65.231, M65.232, M65.239, M65.241, M65.242, M65.249, M65.251, M65.252, M65.259, M65.261, M65.262, M65.269, M65.271, M65.272, M65.279, M65.28, M65.29, M65.30, M65.311, M65.312, M65.319, M65.321, M65.322, M65.329, M65.331, M65.332, M65.339, M65.341, M65.342, M65.349, M65.351, M65.352, M65.359, M65.4, M65.80, M65.811, M65.812, M65.819, M65.821, M65.822, M65.829, M65.831, M65.832, M65.839, M65.841, M65.842, M65.849, M65.851, M65.852, M65.859, M65.861, M65.862, M65.869, M65.871, M65.872, M65.879, M65.88, M65.89, M65.9, M66.20, M66.211, M66.212, M66.219, M66.221, M66.222, M66.229, M66.231, M66.232, M66.239, M66.241, M66.242, M66.249, M66.251, M66.252, M66.259, M66.261, M66.262, M66.269, M66.271, M66.272, M66.279, M66.28, M66.29, M66.30, M66.311, M66.312, M66.319, M66.321, M66.322, M66.329, M66.331, M66.332, M66.339, M66.341, M66.342, M66.349, M66.351, M66.352, M66.359, M66.361, M66.362, M66.369, M66.371, M66.372, M66.379, M66.38, M66.39, M66.80, M66.811, M66.812, M66.819, M66.821, M66.822, M66.829, M66.831, M66.832, M66.839, M66.841, M66.842, M66.849, M66.851, M66.852, M66.859, M66.861, M66.862, M66.869, M66.871, M66.872, M66.879, M66.88, M66.89, M66.9, M67.00, M67.01, M67.02, M67.311, M67.322, M67.331, M67.332, M67.341, M67.351, M67.352, M67.371, M67.372, M67.50, M67.51, M67.52, M67.80, M67.811, M67.812, M67.813, M67.814, M67.819, M67.821, M67.822, M67.823, M67.824, M67.829, M67.831, M67.832, M67.833, M67.834, M67.839, M67.841, M67.842, M67.843, M67.844, M67.849, M67.851, M67.852, M67.853, M67.854, M67.859, M67.861, M67.862, M67.863, M67.864, M67.869, M67.871, M67.872, M67.873, M67.874, M67.879, M67.88, M67.89, M67.90, M67.911, M67.912, M67.919, M67.921, M67.922, M67.929, M67.931, M67.932, M67.939, M67.941, M67.942, M67.949, M67.951, M67.952, M67.959, M67.961, M67.962, M67.969, M67.971, M67.972, M67.979, M67.98, M67.99, M70.031, M70.032, M70.039, M70.10, M70.11, M70.12, M70.20, M70.21, M70.22, M70.30, M70.31, M70.32, M70.40, M70.41, M70.42, M70.50, M70.51, M70.52, M70.60, M70.61, M70.62, M70.70, M70.71, M70.72, M70.90, M71.00, M71.011, M71.012, M71.019, M71.021, M71.022, M71.029, M71.031, M71.032, M71.039, M71.041, M71.042, M71.049, M71.051, M71.052, M71.059, M71.061, M71.062, M71.069, M71.071, M71.072, M71.079, M71.08, M71.09, M71.10, M71.121, M71.122, M71.129, M71.161, M71.162, M71.20, M71.21, M71.22, M71.30, M71.311, M71.312, M71.319, M71.321, M71.322, M71.329, M71.331, M71.332, M71.339, M71.341, M71.342, M71.349, M71.351, M71.352, M71.359, M71.371, M71.372, M71.379, M71.38, M71.39, M71.40, M71.421, M71.422, M71.429, M71.431, M71.432, M71.439, M71.441, M71.442, M71.449, M71.451, M71.452, M71.459, M71.461, M71.462, M71.469, M71.471, M71.472, M71.479, M71.48, M71.49, M71.50, M71.521, M71.522, M71.529, M71.531, M71.532, M71.539, M71.541, M71.542, M71.549, M71.551, M71.552, M71.559, M71.561, M71.562, M71.569, M71.571, M71.572, M71.579, M71.58, M71.80, M71.811, M71.812, M71.819, M71.821, M71.822, M71.829, M71.831, M71.832, M71.839, M71.841, M71.842, M71.849, M71.851, M71.852, M71.859, M71.861, M71.862, M71.869, M71.871, M71.872, M71.879, M71.88, M71.89, M71.9, M72.2, M75.00, M75.01, M75.02, M75.100, M75.101, M75.102, M75.110, M75.111, M75.112, M75.120, M75.121, M75.122, M75.20, M75.21, M75.22, M75.30, M75.31, M75.32, M75.40, M75.41, M75.42, M75.50, M75.51, M75.52, M75.80, M75.81, M75.82, M76.00, M76.02, M76.10, M76.11, M76.12, M76.20, M76.21, M76.22, M76.30, M76.40, M76.41, M76.42, M76.50, M76.51, M76.52, M76.60, M76.61, M76.62, M76.70, M76.71, M76.72, M76.811, M76.812, M76.819, M76.821, M76.822, M76.829, M76.891, M76.892, M76.899, M77.00, M77.01, M77.02, M77.10, M77.11, M77.12, M77.20, M77.21, M77.22, M77.30, M77.31, M77.32, M77.40, M77.41, M77.42, M77.50, M77.51, M77.52, M77.8, M77.9, M79.601, M79.602, M79.603, M79.604, M79.605, M79.606, M79.609, M79.621, M79.622, M79.629, M79.631, M79.632, M79.639, M79.641, M79.642, M79.643, M79.644, M79.645, M79.646, M79.651, M79.652, M79.659, M79.661, M79.662, M79.669, M79.671, M79.672, M79.673, M79.674, M79.675, M79.676, M89.30, M89.311, M89.312, M89.319, M89.321, M89.322, M89.329, M89.331, M89.332, M89.333, M89.334, M89.339, M89.341, M89.342, M89.349, M89.351, M89.352, M89.359, M89.361, M89.362, M89.363, M89.364, M89.369, M89.371, M89.372, M89.379, M89.38, M89.39, M89.50, M89.511, M89.512, M89.519, M89.521, M89.522, M89.529, M89.531, M89.532, M89.539, M89.541, M89.542, M89.549, M89.551, M89.552, M89.559, M89.561, M89.562, M89.569, M89.571, M89.572, M89.579, M89.58, M89.59, M89.8X0, M89.8X1, M89.8X2, M89.8X3, M89.8X4, M89.8X5, M89.8X6, M89.8X7, M89.8X8, M89.8X9, M89.9, M94.1, M94.261, M94.262, M94.269, M94.352, M94.8X0, M94.8X1, M94.8X2, M94.8X3, M94.8X4, M94.8X5, M94.8X6, M94.8X7, M94.8X8, M94.8X9, M94.9, M99.06, M99.07, Q66.89, Q74.9, R25.2
Headache	G43.001, G43.009, G43.011, G43.019, G43.101, G43.109, G43.109 A, G43.111, G43.119, G43.401, G43.409, G43.411, G43.419, G43.501, G43.509, G43.511, G43.519, G43.601, G43.609, G43.611, G43.619, G43.701, G43.709, G43.711, G43.719, G43.801, G43.809, G43.811, G43.819, G43.821, G43.829, G43.831, G43.839, G43.901, G43.909, G43.911, G43.919, G43.B0, G43.B1, G43.C0, G43.C1, G44.001, G44.009, G44.011, G44.019, G44.021, G44.029, G44.039, G44.049, G44.051, G44.059, G44.099, G44.1, G44.201, G44.209, G44.211, G44.219, G44.221, G44.229, G44.301, G44.309, G44.311, G44.319, G44.321, G44.329, G44.51, G44.52, G44.53, G44.59, G44.81, G44.82, G44.83, G44.84, G44.85, G44.89, M54.81, R51
Urogenital, pelvic, and menstrual pain	A54.22, A59.02, N20.9, N21.0, N21.1, N21.8, N21.9, N22, N30.10, N30.11, N41.0, N41.1, N41.9, N42.0, N45.1, N50.811, N50.812, N50.819, N50.82, N50.89, N50.9, N51, N53.12, N71.0, N71.1, N71.9, N72, N73.0, N73.1, N73.2, N73.3, N73.4, N73.5, N73.8, N73.9, N80.0, N80.1, N80.2, N80.3, N80.4, N80.5, N80.6, N80.8, N80.9, N94.0, N94.10, N94.11, N94.12, N94.19, N94.2, N94.3, N94.4, N94.5, N94.6, N94.810, N94.818, N94.819, N94.89, R10.2
Musculoskeletal chest pain	G58.0, I20.0, I20.1, I20.8, I20.9, M94.0, M99.08, R07.1, R07.2, R07.81, R07.82, R07.89, R07.9, S23.41XA, S23.41XD, S23.41XS, S23.420 A, S23.420D, S23.420S, S23.421 A, S23.421D, S23.421S, S23.428 A, S43.206 A, S43.214 A, S43.214D, S43.214S, S43.215 A, S43.215D, S43.215S, S43.216 A, S43.216D, S43.216S, S43.4
Alcohol abuse/dependence	F10.x, G62.1, 291, 303.x, 305, 305.01–03, 357.5, 425.5, 535.3x, 571.x, v11.3, G31.2, G72.1, K85.2x, K75.81, K70.x, I42.6, K29.x, K85.x, Z71.41
Opioid abuse/dependence	F11x, 304.0x, 305.5x(no return of patients not included in other codes)
